# Construction and Characterization of n6-Methyladenosine-Related lncRNA Prognostic Signature and Immune Cell Infiltration in Kidney Renal Clear Cell Carcinoma

**DOI:** 10.1155/2022/7495183

**Published:** 2022-09-29

**Authors:** Zerong Chen, Zehai Huang, Jialiang Hui, zhuangfei Chen, Haibo Zhang, Yaodong Jiang, Weisen Zeng

**Affiliations:** ^1^Department of Cell Biology, School of Basic Medical Sciences, Southern Medical University, Guangzhou 510515, China; ^2^Department of Urology, Nanfang Hospital, Southern Medical University, Guangzhou 510515, China; ^3^Department of Organ Transplant, Nanfang Hospital, Southern Medical University, Guangzhou 510515, China

## Abstract

**Background:**

Kidney renal clear cell carcinoma (KIRC) lacks effective prognostic biomarkers and the role and mechanism of N6-methyladenosine (m6A) modification of long noncoding RNAs (lncRNAs) in KIRC remain unclear.

**Methods:**

We extracted standard mRNA-sequencing and clinical data from the TCGA database. The prognostic risk model was obtained by Lasso regression and Cox regression. We randomly divided the samples into training and test sets, each taking half of the cases. Based on Lasso regression and Cox regression for training set, the prognostic risk signature was constructed; risk scores were calculated with the R package “glmnet.” Based on the median value of the prognostic risk score, risk scores were calculated for each patient and we divided all KIRC samples into high-risk and low-risk groups. Then, high- and low-risk subtypes were established and their prognosis, clinical features, and immune infiltration microenvironment were evaluated in test set and the entire sampled data set. The reliability of the prognostic model was confirmed by receiver operating characteristic curve analysis.

**Results:**

We found 28 prognostic m6A-related lncRNAs and established a m6A-related lncRNAs prognostic signature. Risk score=*AC*015813.1*∗*(0.0086)+*EMX*2*OS∗*(−0.0101)+*LINC*00173*∗*(0.0309)+*PWAR*5*∗*(−0.0146)+*SNHG*1*∗*(0.0043). The signature showed a better predictive ability than other clinical indicators, including tumor node metastasis classification (TNM), histological, and pathological stages. In the high-risk group, M0 macrophages, CD8+ T cells, and regulatory T cells had significantly higher scores. Contrarily, in the low-risk group, activated dendritic cells, M1 macrophages, mast resting cells, and monocytes had significantly higher scores. In the high-risk group, LSECtin was overexpressed. In the low-risk group, PD-L1 was overexpressed. Moreover, high-risk patients may benefit more from AZ628.

**Conclusions:**

In conclusion, prognosis prediction of patients with KIRC and new insights for immunotherapy are provided by the m6A-related lncRNA prognostic signature.

## 1. Introduction

Kidney renal clear cell carcinoma (KIRC) is an adenocarcinoma originating from tubular epithelial cells. KIRC is the most aggressive pathological subtype and accounts for more than 70% of all renal cell carcinoma (RCC) cases [1]. Since most patients with KIRC are not sensitive to chemoradiotherapy, surgical operation remains an effective treatment [2, 3]. However, patients with advanced KIRC are prone to relapse after surgery, resulting in a poor prognosis [4–6]. KIRC has significant heterogeneity and complex pathogenesis, making it difficult to delay the progression and metastasis of KIRC as early as possible. Thus, it is important to identify effective potential therapeutic targets. However, it is difficult to improve the diagnostic accuracy and guide the choice of treatment for KIRC patients based on the TNM staging system. Several polygenic risk models have been explored, such as the miRNA model [7], glycolysis-related gene signature [8], and metabolism-related gene signature [9]. There are different advantages and limitations in these risk models; therefore, the establishment of new biomarkers and prognostic models are required for KIRC patients.

Long noncoding RNAs (lncRNAs) do not encode proteins, and the transcription length exceeds 200 nucleotides. LncRNAs regulate approximately 70% of the genetic expression through the regulation of epigenetics, transcription, and post-transcription, including complex physiological and biochemical mechanisms of both normal and cancerous tissues, such as cell proliferation, differentiation, and carcinogenesis [10–14]. According to previous reports, the downregulated or upregulated expression of lncRNAs play their respective roles in cancers, especially in KIRC [15–17]. For instance, the lncRNA FGD5-AS1 was identified to be a potential biomarker of KIRC since it was significantly associated with Von Hippel-Lindau syndrome [18]. Similarly, another study revealed that the lncRNA CDKN2B-AS1 played a carcinogenic role and can serve as a therapeutic target of KIRC [19].

N6-methylandenosine (m6A) modification is characterized by maintaining dynamic change based on “writers” (methylases), “erasers” (demethylases), and “readers” (signal transducers) [20]. According to previous reports, aberrant regulation of m6A resulted in oncogenic activity, driving the malignancy and immunomodulatory abnormalities [21–23]. A study identified that METTL3 promotes KIRC progression through m6A and provides epitranscriptional insights [24]. In addition, METTL14 was reported to be a prognostic protective factor of KIRC [25]. Overall, m6A-modified lncRNAs are widely involved in tumor progression, metastasis, chemoradiotherapy sensitivity, and immune infiltration [21, 26–28]. However, the mechanism of m6A-modified lncRNAs in KIRC remains unclear.

Finally, we constructed a m6A-related lncRNAs prognostic signature and it can provide prognosis prediction of patients with KIRC and new insights for immunotherapy.

## 2. Materials and Methods

### 2.1. Data Source

We extracted standard mRNA-sequencing and clinical data from the TCGA database (https://www.cancer.gov/). 528 KIRC samples and 72 adjacent normal samples were obtained after excluding those with missing survival information.

### 2.2. Bioinformatics and Statistical Analysis

Based on the R package “ballgown,” we obtained the differential lncRNAs between KIRC samples and control and used the above differential lncRNAs for subsequent analysis (with the ∣logFC∣≥ 2 and *p* < 0.05). We obtained m6A-regulatory genes including eight “writers” (RBM15, RBM15 B, VIRMA, WTAP, METTL3, METTL14, METTL16, and ZC3H13), two “erasers” (ALKBH5 and FTO), and 13 “readers” (YTHDC1/2, YTHDF1/2/3, IGF2BP1/2/3, FMR1, HNRNPC, RBMX, HNRNPA2B1, and LRPPRC) [29]. Based on the gene expression matrix of 239 lncRNAs and m6A-regulatory genes, Pearson's correlation analysis was performed with R package “psych” (with the filtering threshold of correlation coefficient >0.5, *p* value < 0.05). We extracted m6A-related lncRNAs (*p* < 0.05). We randomly divided the samples into training and test sets, each taking half of the cases. Based on Lasso regression and Cox regression for the training set, the prognostic risk signature was constructed; risk scores were calculated with the R package “glmnet” [30]. Thus, we identified 28 filtered genes and calculated the risk score for all KIRC patients using the formula:Risk score=∑_*i*=1_^*n*^Coef_*i*_*∗x*_*i*_ (*x*_i_ represents the expression of lncRNAs; Coef_i_ represents the regression coefficient of each lncRNA). Based on the median value of the prognostic risk score, risk scores were calculated for each patient, and we divided all KIRC samples into high-risk and low-risk groups. Then, to evaluate and validate the reliability of the model, we constructed a time-longitudinal receiver operating characteristic (ROC) curve. We then performed survival analysis using the R package “Kaplan–Meier survival”. We evaluated whether the clinical characteristics, including TNM, pathological, and histological stages, were correlated with the risk scores. We analyzed immune infiltration and immune checkpoints including LESCtin and PD-L1 in two risk groups. Ultimately, with the R package “pRRophetic,” signature-related drug sensitivity predictions were conducted [31].

## 3. Results

### 3.1. Screening and Identifying of m6A-Related lncRNAs in KIRC Patients

**Figure 1:**
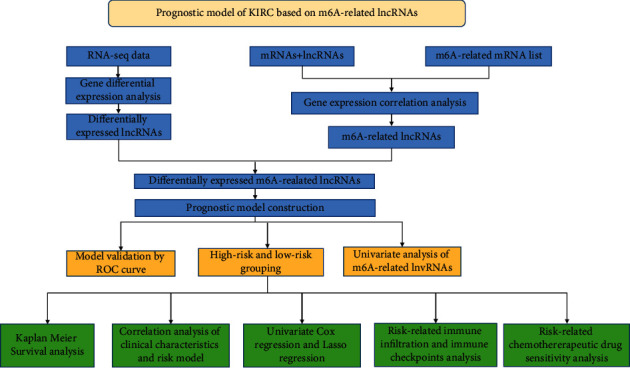
Workflow chart.

**Figure 2:**
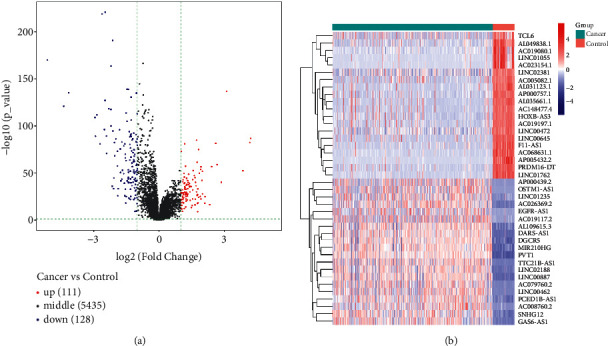
Screening of differentially expressed lncRNAs. (a) Heat map of the first 20 differential lncRNAs in KIRC tumor tissues and normal tissues. The blue bar represents the cancer cases, and the red bar represents the control cases. (b) Volcano map of the differentially expressed lncRNAs. Red dots represent the upregulated lncRNAs and blue dots represent the downregulated lncRNAs.

The study workflow is shown in Figure 1. We downloaded the data of 600 samples, composed of 528 KIRC samples and 72 normal samples, from the TCGA database. Based on the differential analysis, 239 lncRNAs, composed of 111 upregulated cases and 128 downregulated cases, were screened in the KIRC samples. Figures 2(a)) and 2(b) shows the first 20 differential lncRNAs. We also collected 23 m6A-regulatory genes and analyzed the correlation between these regulators and the differential lncRNAs. Finally, 28 prognostic m6A-related lncRNAs were identified.

### 3.2. Construction and Validation of the Prognostic Risk Signature

**Figure 3:**
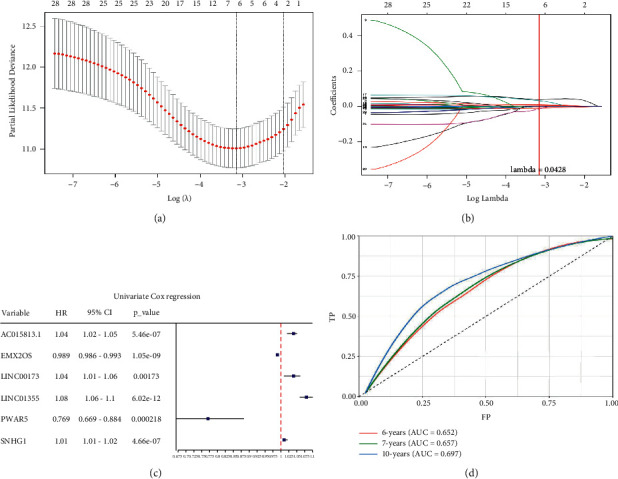
Construction and validation of the Lasso regression signature. (a) The lowest point of the lasso regression curve determines the penalty value. (b) Depending on whether the penalty value intersects each curve, the lncRNAs in the signature were selected and we calculated the regression coefficients. (c) The forest map is constructed based on the univariate Cox regression analysis. (d) The area under the curve (AUC) value of the signature at different prediction endpoints using the receiver operating characteristic (ROC) curve.

Univariate Cox regression was used to identify prognosis-related lncRNAs. Lasso regression with 10-foldcross-validation was performed as shown in Figure 3(a)–3(b) (*λ *min = 0.0428). The prognostic signature contained six lncRNAs, namely AC015813.1, EMX2OS, LINC00173, LINC01355, PWAR5, and SNHG1 (Figure 3(c)). Risk score=*AC*015813.1*∗*(0.0086)+*EMX*2*OS∗*(−0.0101) +*LINC*00173*∗*(0.0309)+*P*WAR5*∗*(−0.0146)+*SNHG*1*∗*(0.0043). A positive coefficient indicated that it was a risk factor, whereas a negative coefficient indicated a protective factor in KIRC. Then, we calculated the area under the curve (AUC) value of the ROC curve in the signature at different prediction endpoints (Figure 3(d)). The results indicated that overexpressed EMX2OS and PWAR5 were associated with a better prognosis in KIRC patients, while the higher expression levels of AC015813.1, LINC00173, LINC01355, and SNHG1 were correlated with a worse prognosis. Moreover, the AUC value of the prognosis model for the overall survival of 6, 7 and 10 years can reach more than 0.6, indicating that the prediction effect of the model is ideal.

### 3.3. Prognostic Value of the Signature

**Figure 4:**
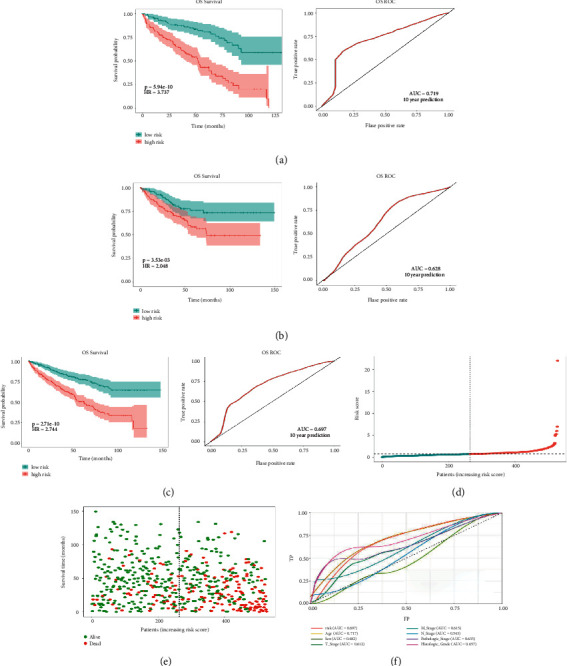
Differences in prognosis between the high-risk and low-risk groups based on the prognostic signature. (a) Kaplan–Meier survival analysis in the training set and the 10-year ROC curves. (b) Kaplan–Meier survival analysis in the testing set and the 10-year ROC curves. (c) Kaplan–Meier survival analysis in the total cases and the 10-year ROC curves. (d) Risk score distribution of patients in the high-risk and low-risk groups. (e) Overall survival status distribution of KIRC patients with increased risk score. (f) The signature has a more favorable clinical value than the T stage, M stage, N stage, histological grade, and pathologic stage by ROC curve analysis.

**Figure 5:**
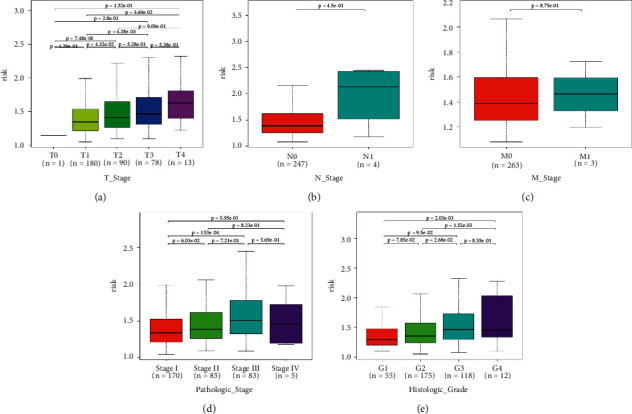
(a–e) Risk scores of patients in the T stage, N stage, M stage, pathological stage, and histological grade differ by stage.

In the high-risk group, patients had a worse prognosis (Figures 4(a)–4(c)). The corresponding ROC curves were obtained, and the AUC was 0.697 for the prognostic risk signature. The deaths of KIRC patients increase with increasing risk (Figures 4(d)–4(e)). In addition, we compared the AUC values between the risk model and other clinical indicators, including TNM, histological, and pathological stages (Figure 4(f)). As a result, the signature showed better predictive ability. After scoring based on the signature, we divided the patients according to the TNM stage, pathological stage, and histological grade and compared the risk differences. The risk scores did not differ significantly (stage M0/1; N0/N1) due to the small number of samples in groups M1 (*n* = 3) and N1 (*n* = 4). The risk scores of stage T2/3/4 were generally higher than those of stage T1. The risk scores of pathological stage II/III were higher than those of pathological stage I. Similarly, the risk scores of G2/3 were higher than those of G1 based on histological grade. The above results are shown in Figures 5(a)–5(e).

### 3.4. Infiltration Abundances of Immune Cells of Two Risk Groups

**Figure 6:**
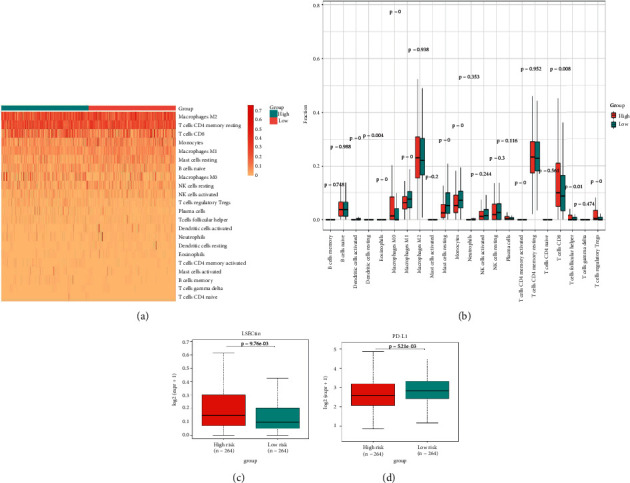
Correlations between the risk scores and infiltration abundances of immune cells and immune checkpoints. (a) Heat map of immune infiltration cells between the high-risk and low-risk groups. (b) Box plot of immune infiltration between n the high-risk and low-risk groups. (c) Differential LSECtin expression in the high-risk and low-risk groups. (d) Differential PD-L1 expression in the high-risk and low-risk groups.

A heat map was created to explore infiltration abundances of immune cells of the two risk groups based on CIBERSORT (Figure 6(a)). In the high-risk group, M0 macrophages, CD8+ T cells, and regulatory T cells had higher scores (Figure 6(b)). Activated dendritic cells, M1 macrophages, mast resting cells, and monocytes account for a larger proportion in the low-risk group. We checked immune checkpoint genes in the two groups to investigate whether there was a significant correlation. In the high-risk group, LSECtin was overexpressed (Figures 6(c)–6(d)). In the low-risk group, PD-L1 was overexpressed.

### 3.5. Drug Sensitivity Prediction Based on the Risk Model

In addition to immune checkpoint blocking therapy, we referred to the Cancer Genomic Project database and identified 94 KIRC-related drugs. The IC50 of these drugs was analyzed and high-risk patients may benefit more from AZ628 (supplementary material 1).

## 4. Discussion

LncRNAs are crucial in the development, progression, dissemination, and treatment resistance of genitourinary malignancies. First, lncRNAs are involved in the main carcinogenic events of genitourinary malignancies, including androgen receptor signaling and hypoxia-inducible factor pathway activation [32, 33]. LncRNAs are also key regulatory factors in the colonization and intravascular transit of cancer metastasis [34]. In addition, lncRNAs are considered essential epigenetic regulators that modulate key molecules in cancer treatment and drug resistance [35, 36]. Thus, lncRNAs are promising candidates for diagnostic markers, therapeutic targets, and prognostic factors in KIRC. With its integral role in modulating diverse physiological processes, especially in tumor microenvironment remodeling, m6A modification may be utilized in therapeutic interventions against cancer [37–39]. A previous study investigated the METTL14/BPTF axis in RCC to reveal the integral role of m6A modification [40].

In the current study, we have identified a novel prognostic signature in KIRC patients. Overexpressed EMX2OS and PWAR5 were associated with a better prognosis in KIRC patients, while the higher expression levels of AC015813.1, LINC00173, LINC01355, and SNHG1 were correlated with a worse prognosis. EMX2OS, as an antisense transcript from EMX2 in the urogenital system [41], was reported to be a metabolism-associated and prognostic protective lncRNA for KIRC [42]. In other studies, EMX2OS was also identified as a protective prognostic index for KIRC patients, including a signature of four hypoxia-associated lncRNAs [16] and a glycolysis-based lncRNA signature [43]. Moreover, one study indicated that EMX2OS is a biomarker for renal allograft survival [44]. Another study reported that EMX2OS acted as a synergistic role in regulating the proliferation and migration of prostate cancer cells [45]. In contrast, little research has been conducted on the basic information and biological functions of the Prader Willi/Angelman region RNA 5 (PAR5; also known as PWAR5). The PAR proteins participated in apoptosis and stress response to cancers [46]. A study proposed that PAR5 prevented premature mitotic entry by modulating CDK-1 phosphorylation [47]. In human tumors, PAR5 is a tumor suppressor in anaplastic thyroid carcinomas [48] and gliomas [49]. In general, the two lncRNAs mentioned above have been widely confirmed as protective factors in published studies. However, the role of AC015813.1 in KIRC remains unknown. As for LINC00173, its high expression promoted the progression of cervical cancer by targeting miR-3171 [50], hepatocellular carcinoma via the microRNA-641/RAB14 axis [51], triple-negative breast cancer [52], glioma through sponging miR-765 [53], colorectal cancer by regulating the miR-765/PLP2 axis [54], and lung squamous cell carcinoma by sponging miR-511-5p to regulate VEGFA expression [55]. In other studies, LINC00173 was reported to inhibit pancreatic cancer by repressing sphingosine kinase 1 protein expression [56]. In conclusion, a considerable number of studies revealed its prognostic role in a variety of cancers. Interestingly, in two independent studies on cervical cancer [50, 57], LINC00173 plays a dual role as a promoter and inhibitor, which may be attributed to the different biological functions it targets. Another study indicated that it serves as a sponge for miR-338-3p to promote prostate cancer progression by regulating Rab25 [58]. However, there are no relevant reports on RCC in general. LINC01355 was reported to be a tumor suppressor in breast cancer [59] and a tumor enhancer in gastric cancer [60] and oral squamous cell carcinoma [61]. The regulatory mechanisms of SNHG1 in cancers have been widely studied. It was reported that SNHG1 activated STAT3 and PD-L1 to regulate the immune escape of RCC [62]. Moreover, SNHG1 negatively regulated miR-137 to promote RCC progression and metastasis [63].

Previous studies have shown that the prognosis of KIRC worsens with an increase in the pathological stage [64]. Clear cell RCC was more often of higher grade and advanced TNM stages than papillary RCCs [65]. Consistently, in the current study, there was a worse prognosis for KIRC patients of higher grades (G2+G3) and advanced T stage.

Although KIRC is susceptible to immunotherapy, the tumor microenvironment (TME) of RCC contains a relatively unique level of immune infiltration [66]. Higher levels of tumor CD8+ T cell infiltration suggest a worse prognosis in KIRC [66, 67]. In our study, CD8+ T cells accounted for a larger proportion in the high-risk group. In the low-risk group, activated dendritic cells had significantly higher scores. A new specific immunotherapeutic approach, such as Rocapuldencel-T autologous immunotherapy for KIRC patients, involves dendritic cell vaccination against cancer [68–70]. In the high-risk group, M0 macrophages had significantly higher scores. In the low-risk group, M1 macrophages had significantly higher scores. A study indicated that patients with high infiltration of M1 macrophages may benefit more from ICI therapy, while high infiltration of M0 macrophages will have the opposite effect [71]. In the high-risk group, regulatory T cells had significantly higher scores. Regulatory T cells are regarded as inhibitors of antitumor immunity, and their elimination may augment natural and pharmacologic immunity [72], which is corroborated herein. Taken together, the level of immune infiltration in KIRC patients seems to be different from that in other forms of cancer. Notably, in the low-risk group, PD-L1 was significantly overexpressed. It is thus necessary to develop new anti-PD-1/PD-L1 agents to treat KIRC [67, 73]. Moreover, we can predict the sensitivity of chemotherapy drugs based on the differences in IC50.

This study had some limitations. First, further verification of experimental and clinical studies will be beneficial. Furthermore, the specific regulatory mechanisms and signaling pathways in which m6A-related lncRNAs interact with immune infiltrating cells in regulating the tumor immune microenvironment require further investigation. Nevertheless, despite these limitations, we identified m6A-related lncRNAs and explored cell-infiltrating characteristics of two risk groups based on the risk model, which can be used to guide immunotherapy.

In conclusion, we developed a prognostic signature in patients with KIRC. Moreover, this prognostic signature was associated with immune checkpoints and immune infiltrating cells, providing novel potential targets to guide immunotherapy in KIRC.

## Data Availability

All data generated or analyzed during this study are included in this published article and the data supporting the findings of this study are available from the corresponding author upon request.

## Supplementary Materials

Supplementary Materials Supplementary material 1. The figure shows the drug sensitivity prediction based on the risk model and the high-risk group is more sensitive to AZ628.
