# Supplementary material for: Construction and Characterization of n6-Methyladenosine-Related lncRNA Prognostic Signature and Immune Cell Infiltration in Kidney Renal Clear Cell Carcinoma
Source: J Oncol. 2022 Sep 29;2022:7495183. doi: 10.1155/2022/7495183 (PMC9536954; doi:10.1155/2022/7495183)
Supplement: Supplementary Materials — Supplementary material 1. The figure shows the drug sensitivity prediction based on the risk model and the high-risk group is more sensitive to AZ628. [file 7495183.f1.docx]

# Supplementary Description

Supplementary material 1. The figure shows the drug sensitivity prediction based on the risk model and the high-risk group is more sensitive to AZ628.
